# Acknowledgement to Reviewers of *Viruses* in 2016

**DOI:** 10.3390/v9010008

**Published:** 2017-01-11

**Authors:** Viruses Editorial Office

**Affiliations:** MDPI AG, St. Alban-Anlage 66, 4052 Basel, Switzerland; viruses@mdpi.com

The editors of *Viruses* would like to express their sincere gratitude to the following reviewers for assessing manuscripts in 2016.

We greatly appreciate the contribution of expert reviewers, which is crucial to the journal’s editorial process. We aim to recognize reviewer contributions through several mechanisms, of which the annual publication of reviewer names is one. Reviewers receive a voucher entitling them to a discount on their next MDPI publication and can download a certificate of recognition directly from our submission system. Additionally, reviewers can sign up to the service Publons (https://publons.com) to receive recognition. Of course, in these initiatives we are careful not to compromise reviewer confidentiality. Many reviewers see their work as a voluntary and often unseen part of their role as researchers. We are grateful to the time reviewers donate to our journals and the contribution they make.

If you are interested in becoming a reviewer for *Viruses*, see the link at the bottom of the webpage http://www.mdpi.com/reviewers.

The following reviewed for *Viruses* in 2016:

Adamson, CatherineHahm, BumsukPerilla, Juan RobertoAdelman, Zachary N.Halary, FranckPerumal, VivekanandanAdhya, SankaHale, BenPeterschmitt, MichelAdler, BarbaraHall, RoyPetrini, StefanoAggarwal, RakeshHalstead, ScottPetrovsky, NikolaiAkari, HirofumiHammerl, Jens-AndrePeyrefitte, ChristopheAlabi, Olufemi J.Hamprecht, KlausPfeiffer, JulieAlbariño, CesarHaralambieva, Iana H.Piconese, SilviaAlbrecht, Randy A.Hardies, Stephen C.Piedra, Pedro A.Alejo, AlíHare, StephenPineyro, Pablo E.Allander, TobiasHarrich, DavidPique, ClaudineAlmazan, FernandoHarrison, RobertPischke, SvenAlonso, CovadongaHartung, John StephenPise-Masison, Cynthia A.Alonso, Marta M.Harvey, RuthPitt, DavidAlvarado, Julia BéjarHatoum-Aslan, AsmaPizzato, MassimoAlves, Ada M. B.Hause, Ben A.Plattet, PhilippeAmarasinghe, GayaHawley, Robert J.Plemper, RichardAmonsin, AlongkornHayasaka, DaisukePoehlmann, StefanAmorim, Maria JoãoHayes, SidneyPoehlmann, StefanAndersen, Kristian G.He, QigaiPolenova, TatyanaAndersson, BjornHearing, PatrickPope, WelkinAranda, Miguel A.Heaton, NicholasPorto, BárbaraArav-Boger, RavitHeckel, GeraldPower, Ultan F.Ariën, Kevin K.Heidari, MohammadPratelli, AnnamariaArnold, MichelleHemmatzadeh, FarhidPrescott, JosephArruda, Bailey L.Hengrung, NarinPronost, Stephane L.Arts, EricHensley, ScottPurcell, MaureenAsgari, SassanHirsch, MatthewPurdy, MikeAshour, JosephHobson-Peters, JodyQin, ZhiqiangAswad, AmrHoeben, RobQiu, BaoliAswad, AmrHoffmann, BerndQiu, Hua-JiAwasthi, SitaHogenhout, SaskiaQiu, XiangguoBabiuk, ShawnHolbrook, MichaelQualley, DominicBabiuk, SteveHolguín-Peña, Ramón JaimeQuax, TessaBailey, Adam LeeHorimoto, TaisukeQuesada, ErnestoBaines, JoelHorvat, BrankaRabkin, CharlesBaize, SylvainHowell, StephenRajendran, RajeswaranBalfour, HenryHu, JianmingRakotondrafara, AurelieBalistreri, GiuseppeHu, QinxueRall, Glenn F.Balkema-Buschmann, AnneHuebbers, Christian U.Randall, Richard EdwardBamford, DennisHussey, GiselaRasmussen, Thomas BruunBanerjee, ManidipaHutchinson, EdwardRatia, KiiraBanfield, BruceIkegami, TetsuroRatner, LeeBanks, LawrenceIlardi, VincenzaRavetch, Jeffrey V.Barbeau, BenoitImbert, IsabelleReading, Patrick C.Barcena, JuanIorio, Ronald M.Rebl, AlexanderBarklis, EricIskra-Caruana, Marie-LineReeves, MatthewBarr, IanIvanov, PavelReguera, Juan J.Barr, JeremyIzumiya, YoshihiroReid, StevenBarrangou, RodolpheJackson, AlanRein, AlanBarrett, Alan D. T.Jackson, WilliamReynolds, MaryBartenschlager, RalfJacquet, StéphanRibeiro, BergmannBeasley, DavidJan, EricRichardson, Christopher D.Beatty, P. RobertJarosinski, Keith W.Richert-Pöggeler, KatjaBecher, PaulJehle, JohannesRicht, JuergenBeer, MartinJensen, KirstyRima, BertBell, AndrewJenson, VictoriaRisatti, GuillermoBellon, MarciaJohne, ReimarRisco, CristinaBelov, GeorgeJohnson, Karyn N.Rivas, C.Benfield, CamillaJohnson, NicholasRivera-Bustamante, RafaelBergelson, JeffreyJohnson, Reed F.Rizvi, TahirBerkhout, BenJones, ClintonRizzo, RobertaBernacchi, SerenaJones, KathrynRodríguez, FernandoBerndsen, Christopher E.Jonsson, Colleen B.Rodríguez, MartaBertoni, GiuseppeJourno, ChloéRodríguez-Lázaro, DavidBickerton, EricaJung, KwonilRomalde, JesusBidgood, SusannaJunglen, SandraRomanowski, VictorBlacklaws, BarbaraKalejta, RobertRömer-Oberdörfer, AngelaBlair, Calor D.Kamar, NassimRoper, Rachel L.Blanchard, YannickKamili, SaleemRoques, PierreBlanco-Lobo, PilarKamitani, WataruRose, Timothy M.Bloom, DavidKanematsu, S.Rosen, Hugo R.Bloom, MarshallKariwa, HiroakiRossi, ShannanBodem, JochemKashanchi, FatahRossman, JeremyBodily, JasonKaufer, BenediktRota, PaulBogner, ElkeKhan, MazharRotenberg, DorithBohn, ErwinKhatri, MaheshRothenburg, StefanBonnefoy, ElietteKhromykh, AlexanderRowlands, David J.Bonning, BryonyKilcher, SamuelRoy, ChadBopegamage, ShubhadaKim, Ki-HongRuggeri, Franco MariaBorca, ManuelKim, Kyun-HwanRuiz, TeresaBoris-Lawrie, KathleenKindrachuk, JasonRümenapf, Hans TillmannBoscia, DonatoKitchen, AndrewRuprecht, KlemensBose, SayantanKlimstra, William B.Ruprecht, Ruth M.Bosse, Jens B.klingstrom, jonasRussell, CharlesBossis, IoannisKlose, ThomasRůžek, DanielBouloy, MichèleKlupp, BarbaraSaalmüller, ArminBowden, Thomas A.Koch, GuusSabanadzovic, SeadBowen, RichardKochneva, GalinaSagan, SelenaBrassard, JulieKochs, GeorgSaijo, MasayukiBrault, AaronKoelle, David M.Sáiz, Juan-CarlosBrennan, BenjaminKogut, Michael H.Sambhara, SuryaprakashBriant, LaurenceKohl, ClaudiaSamulski, JudeBrien, JamesKondabagil, KiranSandmeyer, SuzanneBriers, YvesKondo, HidekiSandri-Goldin, Rozanne M.Brighty, DavidKorennykh, AlexeiSantos, SilvioBrindley, MelindaKousoulas, Konstantin G.Saponari, MariaBrinton, MargoKreher, FelixSargueil, BrunoBroder, ChristopherKrenz, BjörnSarid, RonitBroussard, GregoryKress, AndreaSariyer, IlkerBrown, JayKrishna, NeelSarkar, Mamta ChawlaBrown, JulianneKuhn, JensSarnow, PeterBrown, KatherineKukimoto, IwaoSatou, YorifumiBrugnera, EnricoKumar, SachinSattar, SampurnaBrunotte, LindaKunz, StefanSauleda, SilviaBuchholz, Christian J.Kuroda, Shun’ichiSauter, DanielBuck, Christopher B.Kydd, JuliaSavenkov, EugeneBujarski, JozefLacomme, ChristopheSchaaper, Roeland M.Bukreyev, AlexanderLaflamme, MarKScheel, TroelsBuller, MarkLafon, MoniqueScherrer, Alexandra U.Burri, Dominique JulienLaliberte, Jean-francoisScheuermann, Richard H.Buskiewicz, Iwona A.Lamarre, DanielSchindler, MichaelButtmann, MathiasLambrecht, BénédicteSchlaitz, Anne-LoreCaeiro, MariaLanglois, RyanSchmidt, Aaron GregoryCaffrey, MichaelLauer, Ulrich M.Schneider, DavidCalzolari, MattiaLaurini, ErikSchnell, MatthiasCambillau, ChristianLe Mercier, PhilippeSchnettler, EstherCampos, Samuel K.Lee, BenhurSchols, DominicCao, DianjunLegrand-Abravanel, FlorenceSchönrich, GüntherCaputo, AntonellaLeGrice, StuartSchountz, TonyCarbonell, AlbertoLeib, David A.Schreiner, SabrinaCarocci, MargotLeis, Jonathan P.Schulz, ThomasCarr, JillLeitão, AlexandreSchwemmle, MartinCarr, John P.Lemasson, IsabelleScobie, LindaCarrasco, AdrianoLemiere, StephaneSebastian, SarahCarter, Carol A.LePendu, JacquesSekine, KenTaroCaruso, ClaudioLeppard, KeithSerra-Moreno, RuthCasey, JohnLeticia, Cedillo BarrónServiddio, GaetanoCassar, OlivierLeung, DaisyShaila, M.S.Cattaneo, RobertoLévêque, NicolasShannon-Lowe, ClaireCavassini, MatthiasLevraud, Jean-pierreShatters, RobertChabannes, MatthewLhomme, SébastienSheehy, AnnChan, Chia JungLi, FengSheehy, NoreenChan, Shiu-WanLi, YizeShembade, NoulaChandran, BalaLi, ZejunSherer, NathanChang, Fang-RongLiang, ChenShimizu, KentaChang, JinhongLiang, ZhenglunShimotohno, KunitadaChapman, Nora M.Lieberman, Paul M.Simmons, GregChejanovsky, NorLin, Na-ShengSimon, AnneChen, GuoxunLin, Yi-LingSinclair, JohnCheng, Wei-chiehLindenbach, BrettSingh, Kumud K.Chevaliez, StephaneLingappa, JaisriSironen, TarjaChiou, Shyan-SongLinial, Maxine L.Skalsky, RebeccaCho, Tae-JuLippé, RogerSlagle, Betty L.Choi, Kang-SeukLipton, Howard L.Slama-Schwok, AnnyChoi, Seung-KookLittlejohn, MargaretSloan, ChantelCiminale, VincenzoLiu, SijunSluis-Cremer, NicolasCingolani, GinoLivieratos, Ioannis C.Smagghe, GuyCitovsky, VitalyLizano Soberón, MarcelaSmit, Jolanda M.Cohen, Éric A.Lloyd, RichardSmith, ClintColberg-Poley, AnamarisLo, SzechengSmith, DonaldCompans, RichardLocarnini, StephenSmith, JessicaConrad, NicholasLoc-Carrillo, CatharineSmyth, RedmondConzelmann, Karl-KlausLocker, NicolasSohn, SeonghyangCooper, ChristopherLockhart, BenhamSolorzano, AliciaCorcoran, JenniferLodmell, J. StephenSoloveva, VeronicaCortez, ValerieLofgren, Eric T.Sominskaya, IrinaCosset, François-LoïcLombardi, Vincent C.Spearman, PaulCostello, DeirdreLoo, Yueh-MingSpiegel, MartinCoutts, RobertLopez De Diego, MartaSpiropoulou, Christina F.Crawford, Sue EllenLópez-Moya, Juan JoséSpyrakis, FrancescaCrepin, ThibautLortat-Jacob, HuguesSrinivasan, RajagopalbabCrespo, Mariano SánchezLossius, AndreasStaats, Herman F.Crump, ColinLou, ZhiyongStahelin, Robert V.Cuestas, María LujánLowen, AniceStamminger, ThomasCusi, Maria GraziaLu, ChunStanton, RichardD’Apice, LucianaLu, MengjiStapleford, KennethDąbrowska, KrystynaLundkvist, ÅkeStedman, KennethDallmeier, KaiLünemann, Jan D.Steel, Laura F.Dar, ArshudLuo, HonglinSteinmann, EikeDas, KalyanLy, HihnStern-Ginossar, NoamDawe, Angus L.MacDiarmid, RobinStewart, Cameron R.De Andrés Cara, Damián F.Macdonald, AndrewStonehouse, NicolaDe Berardinis, PiergiuseppeMacFarlane, StuartStoye, Jonathan P.De Grazia, SimonaMackow, Erich R.Strandin, TomasDe Ory, FernandoMaeda, NaoyoshiStraub, TimothyDe Swart, RikMaekawa, ShinyaStrebel, KlausDebes, José D.Mahieux, RenaudStröh, LuisaDelhon, GustavoMahony, Timothy JohnStrong, JamesDelwart, EricMakeyev, EugeneSu, LishanDeng, XufangMakino, ShinjiSullivan, ChrisDeptuła, WiesławMalchiodi, Emilio L.Summerfield, ArturDesbiez, CécileMaliogka, VarvaraSusta, LeonardoDesselberger, UlrichMansky, LouisSutter, GerdDevaux, PatriciaMarcello, AlessandroSuzuki, NobuhiroDewitte-Orr, StephanieMarchant, DavidSuzuki, TetsuroDiaz, ArturoMartel, AnSuzutani, TatsuoDiefenbach, Russell J.Martin Hernandez, Ana M.Svenningsen, Sine LoDietzschold, BernhardMartín, Carmen SanSwitzer, WilliamDigard, PaulMartín, VerónicaSymer, David E.Dikeakos, JimmyMartínez-Salas, EncarnaTagaya, YutakaDimaio, DanielMarzano, ShinyiTakada, AyatoDing, QiangMathiason, CandaceTakegami, TsutomuDölken, LarsMaumus, FlorianTakimoto, ToruDomier, LesMaurizio, ZazziTaliansky, MichaelDonaire, LiviaMauro, VincentTamori, AkihiroDong, WenliangMayer, JensTan, C. SabrinaDoona, ChristopherMazurek, UrszulaTanaka, YuetsuDorf, Martin E.Mazurov, Dmitriy VTang, HengliDorrington, RosemaryMcArthur, Monica A.Tao, PanDouville, Renée N.McCauley, JohnTarlinton, Rachael E.Dreux, MarleneMcDonald, SarahTarr, AlexanderqDrickamer, KurtMcElroy, AnitaTaylor, GrahamDrillien, RobertMcInerney, GeraldTaylor, TravisDrulis-Kawa, ZuzannaMcInnes, ColinTe Velthuis, AartjanDuBois, RebeccaMedigeshi, GuruprasadTedbury, PhilipDuerkop, BreckMedina Piles, VicenteTelenitsky, AliceDuerksen-Hughes, PenelopeMehle, AndrewTesh, Robert B.Dugdale, BenjaminMelero, José A.Theilmann, DavidDunowska, MagdalenaMena, IgnacioTheriault, StevenDustin, LynnMenachery, VineetThomas, EmmanuelDutch, RebeccaMenéndez-Arias, LuisThomas, JulieDvorak, CherylMercer, JasonThoulouze, Marie IsabelleDyall, JulieMesser, WilliamTignon, MaryleneEbihara, HidekiMetzger, MichaelTiley, LaurenceEggink, DirkMeuleman, PhilipTischer, KarstenEhrhardt, AnjaMeulia, TeaTischler, NicoleEiden, MartinMeyers, CraigTisne, CarineEleouet, Jean-FrançoisMichael, Scott F.Tonetti, Michela G.Enquist, Lynn W.Michiels, ThomasTong, ShupingEnright, MarkMiller, CathyTönjes, Ralf ReinhardErrington-Mais, FionaMiller, Jeffrey S.Tortorella, DomenicoEsteban, MarianoMiner, Jonathan J.Tosato, GiovannaEstes, Mary K.Mir, MohammadTramontano, EnzoFaburay, BontoMire, ChadTrevino, AnaFalk, BryceMochizuki, TomofumiTrobaugh, Derek W.Fang, BingliangModis, YorgoTsai, BillyFang, MinMoelling, KarinTzanetakis, Ioannis E.Fang, ShengyunMorgan, IainUeda, KojiFassati, AribertoMorrey, John D.Ulaeto, DavidFeng, ZongdiMorrison, Lynda A.Upton, ChrisFerguson, BrianMoss, William JohnUstav, MartFernández-Romero, JoséMothes, Walther H.Vaheri, AnttiFerreira, FernandoMougel, MaryleneValles, Steven M.Ferriol, InmaculadaMouland, AndrewVan Den Boomen, DickFirth, Andrew E.Mühlberger, ElkeVan Dyk, Linda F.Fisch, PaulMühldorfer, KristinVan Etten, JamesFischer, Matthias GMulero, VictorianoVan Etten, JamesFischer, MattiasMüller, BarbaraVan Grevenynghe, JulienFischer, WolfgangMüller, Martinvan Hemert, MartijnFisher, Derek J.Münch, JanVan Knippenberg, IngeborgFlemington, Erik K.Munoz-Fontela, CesarVaneechoutte, MarioFlores, RicardoMunster, VincentVanWeyenbergh, JohanFodor, ErvinMurakami, SeishiVanwolleghem, ThomasFonseca, FilomenaMurcia, PabloVarani, LucaFontana, JuanMurooka, ThomasVerma, SubhasFontoura, BeatrizMurtaugh, MichaelVidal, SilviaFooks, TonyMusier-Forsyth, KarinVignuzzi, MarcoForbi, Joseph C.Mutsafi, YaelVile, Richard G.Forlenza, MariaMuylkens, BenoîtVoinnet, OlivierForstova, JitkaMyoung, JinjongVolkman, Loy E.Forterre, PatrickNaffakh, NadiaVon Messling, VeronikaFragkoudis, RennosNagy, PeterVreede, FrankFreed, EricNakahara, Kenji S.Waheed, Abdul A.Freiberg, Alexander N.Nakai, HiroyukiWakimoto, HiroakiFrese, MichaelNakamura, NobuhiroWalsh, DerekFreuling, ConradNakanishi, AkiraWan, HenryFreundt, Eric C.Narayanan, AarthiWang, Chin-TienFriedrich, TomNatarajan, ViswanathanWang, JenniferFuchs, MarcNemerow, GlenWang, JiafuFuentes, AlejandroNeogi, UjjwalWang, Pei GangFukushi, HidetoNeumann, Donna M.Wang, RanranFuruya, YoichiNg, JamesWang, YongjieFütterer, JohannesNicoletti, LoredanaWarfield, KellyGagia, Marta MariaNie, XianzhouWark, PeterGagnon, CarlNielsen, Sandra AbelWatanabe, ToshikiGalindo-Cardiel, Iván JoséNiikura, MasahiroWebby, Richard J.Gallagher, TomNilubol, DachritWeber, FriedemannGallardo, CarminaNissimov, JozefWebster, CraigGallay, PhilippeNoah, JamesWedemeyer, HeinerGallinella, GiorgioNoda, TakeshiWeger, StefanGalyov, EdNorder, HeleneWei, KaifaGanesan, Latha P.Nunberg, JackWeingartl, HanaGanges, LlilianneNyborg, Jennifer K.Weir, DawnGao, FengO’Connor, Christine M.Weissenböck, HerbertGao, GuanxiaObbard, Darren JWernike, KerstinGarbuglia, Anna RosaOchoa Corona, FranciscoWhitfield, AnnaGarcia-Estañ, Luis PerezOgawa, HarukoWhittaker, Gary R.Garmedia, AntonioOkamoto, HiroakiWilkinson, GavinGavioli, RiccardoOkuno, TetsuroWilliams, SusanGeballe, AdamOliver, JonathanWilson, AngusGeering, AndrewOlivier, AliciaWilusz, JeffreyGehring, Adam J.Olson, KennethWintermantel, William M.Gerhauser, IngoOno, AkiraWitkowski, PeterGerlier, DenisOoms, MarcelWöhrl, Birgitta M.Gershwin, LaurelOrtego, JavierWolthers, Katja C.Ghosh, MimiOsorio, Fernando A.Worgall, StefanGiansanti, FrancescoOstrowski, MathiasWren, Brendan W.Gilbertson, Robert L.Overton, E. TurnerWu, TuoqiGillet, NicolasPaeshuyse, JanWylie, Stephen J.Gimenez-Lirola, LuisPager, CaraXi, ZhiyongGinhoux, FlorentPaillart, Jean-ChristopheXia, NingshaoGladue, DouglasPalacios, GustavoXiang, Shi-huaGlaunsinger, Britt A.Palese, PeterXie, QingmeiGoff, StephenPalmarini, MassimoYaegashi, HajimeGoffard, AnnePan, XiaolingYan, FeiGoldberg, TonyParadkar, PrasadYeh, Shiou-HweiGonzález Portal, María E.Pardigon, NathalieYogev, OhadGonzalez-Aseguinolaza, GloriaParent, LeslieYoneyama, MitsutoshiGoo, LeslieParet, Mathews L.Yu, Xiao-FangGottwein, EvaParvez, MohammadYutin, NatalyaGouttenoire, JeromePas, Suzanne D.Zaaijer, Hans L.Grand, Roger J. A.Passarelli, A. LorenaZeiser, RobertGrandvaux, NathaliePatil, Basavaprabhu L.Zhang, XiangminGreber, UrsPattnaik, Asit K.Zhang, YanjinGregoire, MarcPatton, JohnZhang, YoujunGribhoffer, LiborPaul, AnikoZheng, Yong-HuiGroves, IanPavlovic, JovanZheng, Zhi-MingGuix, SusanaPearson, AngelaZheng, Zi-ZhengGulley, Margaret L.Peikert, TobiasZhong, ZhaohuaGuo, HaitaoPeñaflor, Maria FernandaZhu, HuanzhangGuo, JutaoPeng, XuZiebell, HeikoGuy, Paul L.Pepin, MichelZiegelbauer, Joseph M.Haagmans, Bart L.Perez, LaurentZiegler-Graff, VeroniqueHabjan, MatthiasPerez-Gracia, Maria-TeresaZúñiga, SoniaHaertle, SonjaPerez-Gracia, Teresa


